# DeepCausality: A general AI-powered causal inference framework for free text: A case study of LiverTox

**DOI:** 10.3389/frai.2022.999289

**Published:** 2022-12-06

**Authors:** Xingqiao Wang, Xiaowei Xu, Weida Tong, Qi Liu, Zhichao Liu

**Affiliations:** ^1^Department of Information Science, University of Arkansas at Little Rock, Little Rock, AR, United States; ^2^Division of Bioinformatics and Biostatistics, National Center for Toxicological Research, US Food and Drug Administration, Jefferson, AR, United States; ^3^Office of Clinical Pharmacology, Office of Translational Sciences, Center for Drug Evaluation and Research, US Food and Drug Administration, Silver Spring, MD, United States

**Keywords:** AI, causal inference analysis, transformer, NLP, DILI

## Abstract

Causality plays an essential role in multiple scientific disciplines, including the social, behavioral, and biological sciences and portions of statistics and artificial intelligence. Manual-based causality assessment from a large number of free text-based documents is very time-consuming, labor-intensive, and sometimes even impractical. Herein, we proposed a general causal inference framework named DeepCausality to empirically estimate the causal factors for suspected endpoints embedded in the free text. The proposed DeepCausality seamlessly incorporates AI-powered language models, named entity recognition and Judea Pearl's Do-calculus, into a general framework for causal inference to fulfill different domain-specific applications. We exemplified the utility of the proposed DeepCausality framework by employing the LiverTox database to estimate idiosyncratic drug-induced liver injury (DILI)-related causal terms and generate a knowledge-based causal tree for idiosyncratic DILI patient stratification. Consequently, the DeepCausality yielded a prediction performance with an accuracy of 0.92 and an F-score of 0.84 for the DILI prediction. Notably, 90% of causal terms enriched by the DeepCausality were consistent with the clinical causal terms defined by the American College of Gastroenterology (ACG) clinical guideline for evaluating suspected idiosyncratic DILI (iDILI). Furthermore, we observed a high concordance of 0.91 between the iDILI severity scores generated by DeepCausality and domain experts. Altogether, the proposed DeepCausality framework could be a promising solution for causality assessment from free text and is publicly available through https://github.com/XingqiaoWang/https-github.com-XingqiaoWang-DeepCausality-LiverTox.

## Introduction

Causality is the study of the relationship between causes and effects, which is the foundation of almost every scientific discipline to verify hypotheses and uncover underlying mechanisms (Pearl, 2009). Notably, causal inference plays an essential role in medical practices to test scientific theories and decipher the etiology for advancing pharmacovigilance, optimize clinical trial designs, and establish real-world evidence (Naidu, 2013; Mazhar et al., 2020; Zheng et al., 2020; Ho et al., 2021). The conventional way to conduct causal inference relies on randomized controlled trials (RCTs) (Zheng et al., 2020). In randomized clinical trials, the test subjects are randomly assigned to one of two groups: the treated group receiving the intervention (e.g., drug) tested and the control group receiving an alternative (e.g., placebo) treatment. Causality is established if the clinical outcome is statistically significant in the treated group over the control one. However, conducting a randomized clinical trial is time-consuming, labor-intensive, expensive, and sometimes even impractical.

Consequently, there has been growing interest in alternative approaches, such as target trials based on observational data, to improve the causality assessment in real-world applications (Frieden, 2017; Gajra et al., 2020; Hernán, 2021). For example, the U.S. Food and Drug Administration (FDA) released guidance on a real-world evidence (RWE) program to create a framework for evaluating the potential use of RWE to help support the approval of a new indication for a drug already approved under section 505(c) of the FD&C Act or to help support or satisfy drug post-approval study requirements (https://www.fda.gov/science-research/science-and-research-special-topics/real-world-evidence). Under the 21^st^ Century Cures Act, the FDA is mandated to evaluate the potential use of real-world data (RWD) and RWE to support the approval of a new indication for a drug. Draft guidance has been issued to address the generation of RWE, including the utilization of claims and electronic health records (EHRs), two major RWD sources, in support of regulatory decision-making. In addition, the FDA has prioritized the creation of an RWE Data Enterprise (the Sentinel System). An essential part of the initiative is incorporating EHR data from about 10 million individuals into the data infrastructure for FDA active drug safety surveillance (https://www.fda.gov/news-events/fda-voices/fda-budget-matters-cross-cutting-data-enterprise-real-world-evidence).

In the past decade, the generation of EHRs has increased substantially in the U.S., partly due to the Health Information Technology for Economic and Clinical Health (HITECH) Act of 2009, which provided $30 billion in incentives for hospitals and physician practices to adopt EHR systems. Whereas administrative claims data are highly structured, much of the potentially useful information contained within EHRs is unstructured, in the form of laboratory data, visit notes (e.g., narrative descriptions of a patient's signs and symptoms, family history, social history), radiology reports or images, and discharge summaries. EHRs contain rich clinical information and complex relations in the data that may not be fully harnessed using more traditional approaches. The ability of EHRs to generate quality RWE depends on whether we can address the challenge in curating and analyzing unstructured data. In response, FDA seeks to incorporate emerging data science innovations, such as natural language processing (NLP) and machine learning, to establish the organizational framework for ensuring high-fidelity, fit-for-purpose EHR data. To inform the causal inference framework for EHR-based signal detection (hypothesis generating), we will evaluate the emerging approaches that have been proposed or tested.

Accumulated observational data provide tremendous opportunities to promote target trials for causality establishment. Thus, there is an urgent need to develop novel statistical models to effectively estimate causal factors embedded in the extensive free text-based observational data. Artificial intelligence (AI) has made substantial progress in a variety of fields, such as computer vision (O'Mahony et al., 2019), NLP (Liu et al., 2021), speech recognition and generation (Hannun et al., 2014), and decision-making (Shrestha et al., 2019). Despite significant progress in AI, we still face a great challenge in understanding the mechanisms underlying intelligence, including reasoning, planning, and imagination (Schölkopf, 2019). Recent hype of AI-powered language models (LMs) and advanced statistical measures seem to pave a promising way to enhance the ability of AI in reasoning, such as causal inference (Veitch et al., 2020; Wang et al., 2021). In our previous work, we proposed a transformer-based causal inference framework called InferBERT by integrating the A Lite Bidirectional Encoder Representations from Transformers (ALBERT) (Lan et al., 2019) and Judea Pearl's Do-calculus (Wang et al., 2021). The proposed InferBERT has been successfully applied for causality assessment in pharmacovigilance and exemplified estimation of the causal factors related to opioid-related acute liver failure and tramadol-related mortalities in the FDA Adverse Event Reporting System (FAERS) database. However, there is still much space for improvement for InferBERT to facilitate real-world applications. First, the proposed InferBERT has only been used for structure-based data sets (e.g., FAERS), limiting its application in the free text-based corpus. Although we proposed a synthetic approach to transforming the different clinical entities into a sentence-based representation, the performance of the proposed InferBERT in free text needs to be further investigated. Second, domain-specific knowledge was not considered for causal inference, resulting in false positives or introduction of Irrelevant causal factors.

In this study, we proposed a general AI-powered framework called DeepCausality by fusing transformer, named entity recognition (NER), and Judea Pearl's Do-calculus for causal inference from free text-based documents. To demonstrate the validity of the proposed DeepCausality, we employed the LiverTox database (https://www.ncbi.nlm.nih.gov/books/NBK547852/) to estimate the drug-induced liver injury (DILI)-related causal terms and further verified by using the American College of Gastroenterology (ACG) clinical guideline for idiosyncratic DILI (iDILI) (Chalasani et al., 2021). Furthermore, we developed a causal tree based on verified causal DILI terms and utilized it for iDILI patient stratification based on DILI case reports.

## Materials and methods

### DeepCausality overview

**Figure 1:**
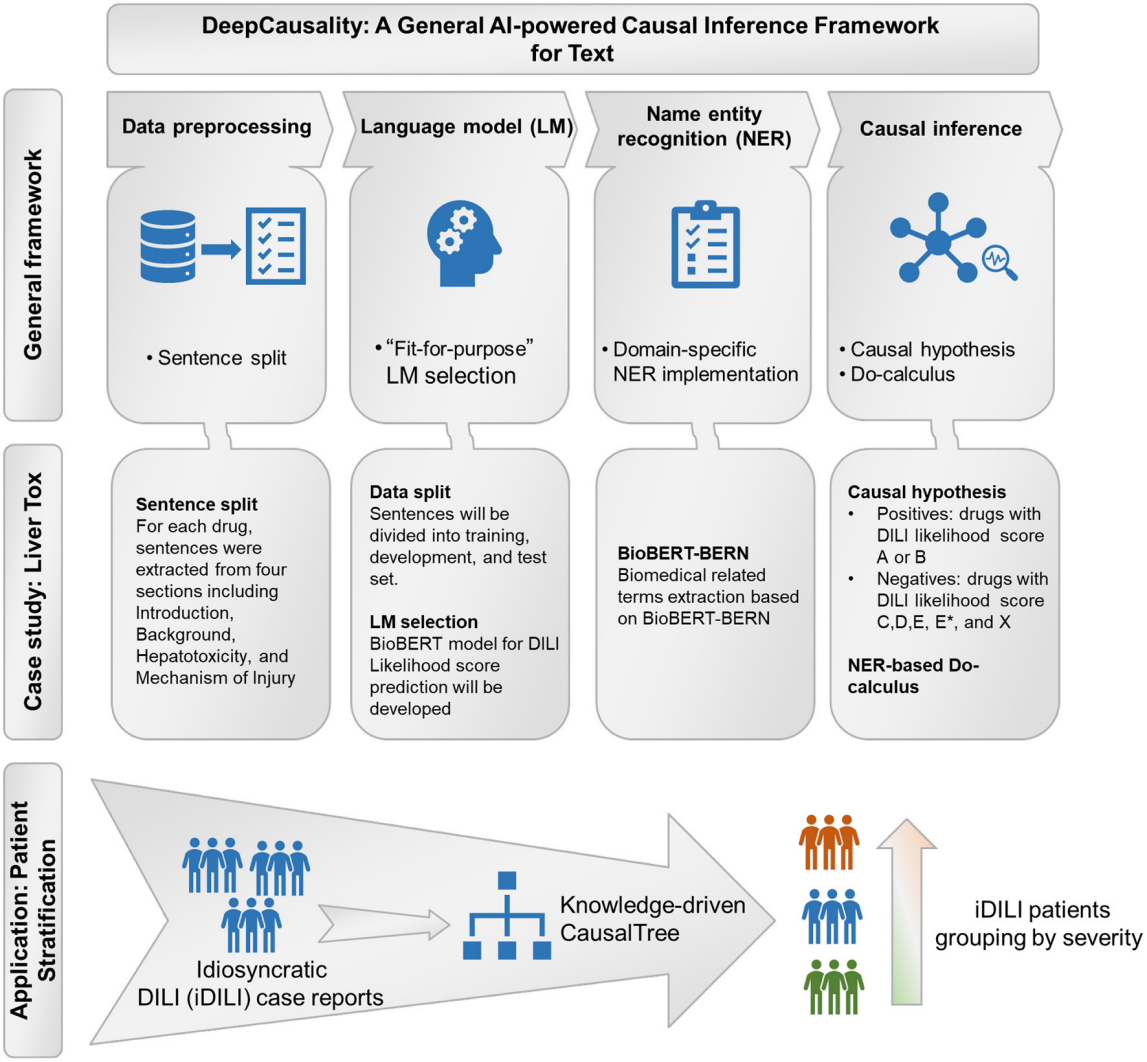
The workflow of the study: General framework of the DeepCausality, case study with LiverTox, and idiosyncratic DILI (iDILI) patient stratification.

The proposed DeepCausality is a general transformer-based causal inference framework for free text, consisting of data preprocessing, LM development, NER, and Do-calculus based causal inference (Figure 1).

#### Data preprocessing

First, the corpus of free text-based documents was split into sentences. Then, an endpoint was assigned to each sentence based on the investigational causal question. For example, suppose you investigate causal factors of lung cancer etiology. The sentences describing the patient with lung cancer and related symptoms and clinical outcomes were labeled as positives, and vice versa. Consequently, we used ***D*** to denote the preprocessed corpus of free text-based documents, where *d*_*i*_ = (*x*_*i*_, *y*_*i*_) ϵ ***D*** indicates the *i-*th instance in the dataset ***D***, *i* = 1,2, …, *N, N* (total number of instances), with *x*_*i*_ (i.e., sentence) and *y*_*i*_ (i.e., endpoint) being the text sequence. We employed *tf-idf* [i.e., term frequency (tf)-inverse document frequency (idf)] values to investigate the distribution of terms in the corpus, which could be calculated based on the below formula,


(1) $$\begin{array}{l} tf-idf\left(t,d\right)=tf{\left(t,d\right)}^{*}idf\left(t\right) \end{array}$$

(2) $$\begin{array}{l} tf\left(t,d\right)=\frac{count\;of\;t\;in\;d}{number\;of\;words\;in\;d} \end{array}$$

(3) $$\begin{array}{l} idf\left(t\right)=log\left(N/\left(df+1\right)\right) \end{array}$$

where *t, d, N* denote term, documents, and number of documents, respectively. The higher *tf-idf* value signified its importance in the document and corpus.

#### Language model development

Conditional probability distribution among words (i.e., tokens) in the text corpus is the basis for causal inference. LM uses various statistical and probabilistic techniques to determine joint probability among the words in the corpus. Specifically, a transformer-based LM could generate all joint probability among tokens as a gigantic probabilistic model using the Masked-Language Modeling (MLM) training strategy, allowing casual assessment among all the variables in the corpus. Two major types of transformer-based LM architectures, Bidirectional Encoder Representations from Transformers (BERT) (Devlin et al., 2018) and its derives (Lan et al., 2019; Liu et al., 2019; Sanh et al., 2019; Clark et al., 2020), and Generative Pre-trained Transformer (GPT) models (Brown et al., 2020), currently dominate the field. Furthermore, efforts have also been made to develop transformers based on the domain-specific corpus [e.g., BioBERT (Lee et al., 2020), ClinicalBERT (Huang and Altosaar, 2019), SciBERT (Beltagy and Lo, 2019), LEGAL-BERT (Chalkidis et al., 2020)] for performance enhancement in specialized domains. Some reports have demonstrated that domain-specific pre-training is a solid foundation for a wide range of downstream domain-specialized NLP tasks (Gu et al., 2021).

With the pre-trained LM, the conditional probability distribution given free text is estimated by the LM-based downstream task. The pre-trained LM computes the attention between tokens. Then, the classification ([CLS]) special token representing the semantic information of the whole sequence is fed into the input layer of the downstream classification model. The softMax layer is adopted as the output layer to access the conditional probability distribution. We use the following cross entropy loss function for the classification of input text sequences:


(4) $$\begin{array}{l} LOSS(D)=-\sum_{1}^{N}(y_{i}*log\left(p\left(x_{i}\right)\right)\\ \;+\left(1-y_{i}\right)*log(1-p(x_{i}))),\;i=1,2,\ldots ,\;N \end{array}$$

where *p*(*x*_*i*_) is the output of the classification model for text sequence *x*_*i*_, which is a calculated probability of the predicted class of *x*_*i*_. *y*_*i*_ is the ground truth label of *x*_*i*_.

By training the classifier with dataset ***D***, we can estimate the conditional probability distribution ***P***(*endpoint*|***X***), where training dataset ***X*** = {*x*_1_, *x*_2_, …, *x*_*N*_}. Then, we use the model to predict all the text sequences for each instance in the dataset ***D***. We denote the output of the classifier as *p*(*x*_*i*_), where *p*(*x*_*i*_) is the probability of the endpoint presented for instance *d*_*i*_.

#### Name entity recognition

According to the task field, our framework adopts a domain-specific NER method, a text mining technique, to extract the name entities in the free text. The NER method can predict the span and category of name entities in the text according to the task with a domain-specific NER method.

For each instance, *d*_*i*_ in dataset ***D***, the NER method recognizes all the name entities in the text sequence *x*_*i*_. Then, we get the set of name entities ***ner***_*i*_ corresponding to *x*_*i*_, where ***ner***_*i*_= {*ner*__*i*_1_, *ner*_*i*_2, …, *ner*_*iM*_}, with *M* being the total number of name entities in the text sequence *x*_*i*_. Next, we combined and unified all the name entities in set ***ner***_***i***_ corresponding to the text in the extracted dataset ***D***. As a result, we obtained the unique name entity set ***NER***, where *NER* = ⋃*ner*_**i**_, *i* = 1, 2, …, *N*; It is the union of ***ner***_***i***_. Then, the recognized name entities were fed into the Do-calculus component of the framework as causal factor candidates.

#### Do-calculus based causal inference

In our previous work, we performed causal inference on structured data by using the Do-calculus mechanism to check whether each feature in the structured data was the cause of the endpoint. In this study, to perform causal inference on the free text, we first extracted name entities in free text and then considered these name entities as causality candidates to infer potential causal factors.

In the proposed framework, the classifier model calculates the conditional probability distribution of the endpoint given the free text sequence. Then, the extracted name entities in each instance sequence act as the endpoint's candidate causal factors. To empirically estimate the candidate name entities in each instance causing the endpoint, we adopted Judea Pearl's Do-calculus framework (Tucci, 2013; Pearl and Mackenzie, 2018).

Do-calculus aims to investigate the interventional conditional probability distribution of ***P***[*endpoint* = true|**DO**(*ner*)] by counterfactually changing the appearance of the name entity *ner*. We use the conditional probability distribution expectation to represent the **DO**(*ner*) and **NOT DO**(*ner*). Suppose there exists a statistically significant difference when comparing the interventional conditional probability distributions of P[endpoint = true|**DO**(ner)] and P[endpoint = true|**NOT DO**(ner)]. In that case, the causality relationship will be established.

Based on the Classification Prediction ***p(*x*_*i*_)*** from the developed classifier, the Do-calculus procedure was performed to estimate the cause of the endpoint. The pseudo-code of the name entity-based Do-calculus procedure is shown below:

**Algorithm 1:** Name entity-based Do-calculus algorithm.


**Input:**Classification Prediction result *p*(*x*), dataset ***D***, ***NER*** results, statistic test threshold *thr* **Output:**Do-calculus results ***C*** 1. set ***C*** = {} // ***C*** is the set of established causes 2. **for** *ner* in ***NER*** **do** // for each name entity 3. set ***S1*** = {} // *S1* contains all results of **DO** (*ner*) 4. set ***S2*** = {} // *S2* contains all results of **NOT DO** (*ner*) 5. **for** *d*_*i*_ in ***D*** **do** // for each instance in the dataset 6. ***S1*** ← *p*(*endpoint*|**DO** (*ner*) // probability of **DO** (*ner*). 7. ***S2***← *p*(*endpoint*|**NOT** **DO** (*ner*) // probability of **NOT DO** (*ner*). 8. z-score = z_test_ (***S1***, ***S2***) // perform z-test based on ***S1*** and ***S2*** 9. **if** z-score > *thr* **then** 10. ***C***←*ner* // ***C*** consists of all established causes 11. **return** ***C***;

For all the extracted name entities, we applied the name entity-based Do-calculus algorithm to check whether it was the cause of the endpoint. For a name entity *ner*, if *ner* ϵ *x*_*i*_, we say instance *d*_*i*_ meets the condition of **DO** (*ner*), while if *ner* 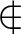 *x*_*i*_, then it doesn't. For *ner*, we assigned the conditional probability *p*[*endpoint*|**DO** (*ner*)] or *p*[*endpoint*|**NOT** **DO** (*ner*)] to sets ***S1*** and ***S2*** respectively. ***S1*** is the set of conditional probability of **DO** (*ner*), while ***S2*** consists of conditional probabilities of those instances NOT DO (*ner*). We used the one tail *z*-test to evaluate whether the probabilities in ***S1*** were significantly different to ***S2***.

We perform one tail *z-*test between S1 and S2. If the *p*-value is less than a threshold like 0.05, we view the *ner* as a cause of the endpoint. To establish all the causal terms of the endpoint, we evaluated every candidate name entity. The generated term set ***C*** is the set of all the name entities that satisfy the statistical significance test.

### Case study: Causal inference of idiosyncratic DILI based on LiverTox

#### Clinical knowledge of idiosyncratic DILI

iDILI is a rare adverse drug reaction, but common in gastroenterology and hepatology practices. The symptoms of iDILI have multiple presentations, characterized from asymptomatic elevations in liver biochemistries to hepatocellular or cholestatic jaundice, liver failure, or chronic hepatitis (Chalasani et al., 2021). Causal factors associated with iDILI recommended by ACG Clinical Guideline could be divided into three types: host, environmental, and drug-related factors (Chalasani et al., 2021). Specifically, host factors include age, gender, pregnancy, malnutrition, obesity, diabetes mellitus, co-morbidities (e.g., underlying liver disease), and indications for therapy. Environmental factors include smoking, alcohol consumption, infection, and inflammatory episodes. Drug-related factors consist of the daily dose, metabolic profiles, class effect and cross-sensitization, and drug interactions and polypharmacy. Furthermore, the ACG clinical guideline also suggested an algorithm to evaluate suspected iDILI by integrating DILI-related clinical measurements and iDILI-associated causal factors (Chalasani et al., 2021).

#### Data preprocessing of the LiverTox database

LiverTox^®^, launched by the National Institute of Diabetes and Digestive and Kidney Diseases (NIDDK) and the National Library of Medicine (NLM), is a DILI atlas dedicated to providing up-to-date, easily accessed information and comprehensive clinical information on iDILI for both physicians and patients (Hoofnagle, 2013). There are 1,095 drug records in the LiverTox database, which are available at https://ftp.ncbi.nlm.nih.gov/pub/litarch/29/31/. For each drug record, the information was organized based on different sections, including Introduction, Background, Hepatotoxicity, Mechanism of Liver Injury, Outcome and Management, Case reports, Chemical and Product Information, and References.

To demonstrate the utility of the proposed DeepCausality framework, we employed drug records stored in the LiverTox^®^ database. The purpose is to use our proposed DeepCausality to estimate the causal factors related to iDILI. For each drug record, we extracted the text from four sections, Introduction, Background, Hepatotoxicity, and Mechanism of Liver Injury, which are the major sections that describe the synthesized knowledge on hepatoxicity. The DILI Likelihood score is embedded in the hepatoxicity section. Each sentence except the one that included the DILI likelihood score in these four sections was considered as *x*_*i*_, and all the extracted sentences were considered as ***D***.

Domain experts developed the DILI likelihood score to categorize drugs based on the likelihood of drugs associated with the known potential of DILI for causing liver injury. The DILI likelihood score is largely opinion-based and derived from published medical literature to categorize the possibility of the drug causing idiosyncratic liver injury, including **Category A** – well known, **Category B** – known or highly likely, **Category C** – probable, **Category D** – possible, **Category E** – not believed or unlikely, and **Category X** – unknown. We labeled each sentence *x*_*i*_ according to the DILI likelihood score. Specifically, if the sentence *x*_*i*_ from the drug with a DILI likelihood score was either Category A or Category B, we assigned the sentence a label *y*_*i*_ as iDILI positives. Otherwise, the sentence was labeled as iDILI negatives.

#### Language model selection

Considering the LiverTox database provided the summarized knowledge on DILI mainly based on medical literature, we selected BioBERT as the domain-specific language model to develop DeepCausality. BioBERT was developed on top of the pre-trained BERT model by further fine-tuning with biomedical-specific corpora, including PubMed abstracts (PubMed) and PubMed Central full-text articles (PMC) using MLM (Lee et al., 2019). BioBERT has shown its superiority in various biomedical-related downstream tasks over the state-of-the-art NLP approaches. To make BioBERT more specific for the DILI application, we further fine-tuned the BioBERT model with the extracted sentences ***D*** from LiverTox. Consequently, the fine-tuned BioBERT could represent the joint conditional probability among words involved in the extracted sentence ***D***.

#### Biomedical entity recognition

Given that many words in the corpus were not biomedical specific, there was the potential risk of bringing false positives during the causal inference process. Therefore, we employed biomedical entity recognition to extract different biomedical-related terms and limit the causal inference within these domain-relevant terms. In this study, we used biomedical entity recognition and a multi-type normalization tool (BERN) to extract biomedical-related terms, including gene/protein, disease, drug/chemical, species information, and genetic variants (Kim et al., 2019). The BERN is a series of BioBERT-named entity recognition models with probability-based decision rules to recognize and discover different biomedical entities, accessible through https://bern.korea.ac.kr. Here, we only considered extracted name entities with more than a frequency of 50 across the corpus as causal factor candidates for further analysis.

#### NER-based Causal inference

The named entity-based Do-calculus strategy was developed to carry out the causal inference within biomedical entities extracted using the BERN. The potential causal terms of iDILI were enriched if the adjusted *p* value was less than 0.05 based on the one-tail z-test calculation. Furthermore, other statistical measures were also provided, including z-score, average DO probability, and average not DO probability.

We further developed a knowledge-based causal tree to organize the enriched causal factors by following the ACG clinical guideline for iDILI diagnosis (see *Clinical knowledge* section). Specifically, the enriched causal terms were classified into different causal factors of iDILI, including Concomitant diseases, History of other liver disorders, Physical findings, Laboratory results, Symptoms and Signs, Clinical outcome, Covering host, Environmental, and Drug-related. Furthermore, the liver enzymes test results were also incorporated into the proposed knowledge-based causal tree to facilitate the iDILI patient stratification.

### Real-world application: Idiosyncratic DILI (iDILI) patient stratification

In the LiverTox database, some drug records contained one or more case reports related to DILI, which were curated from scientific literature or liver-specific clinical databases such as DILI Network (DILIN). The case report comprised the findings from a clinical laboratory, radiologic and histologic testing summarized in a formulaic table titled Key Points, and a short concluding discussion and comment on DILI severity. The key points included iDILI patterns and severity scores, which served as the ground truth for iDILI patient classification. The DILI patterns were divided into three categories (i.e., Hepatocellular - *R* > 5, mixed - 2<*R*<5, and cholestatic - R < 2) by the ratio between serum alanine transaminase (ALT) and aspartate transaminase (AST). The severity score was based on five levels: 1+, Mild; 2+, Moderate; 3+, Moderate to Severe; 4+, Severe; and 5+, Fatal.

Because iDILI is a multifactorial endpoint caused by different underlying mechanisms, it was crucial to stratify iDILI patients into different DILI pattern subgroups to facilitate subsequent treatment regimen development. To demonstrate whether the developed knowledge-based causal tree could be utilized to categorize the iDILI patients, we extracted a total of 175 case reports from LiverTox for further analysis. First, we classified the patients by extracting the causal factors involved in the developed knowledge-based causal tree from each case report. Second, we verified the iDILI patient stratification results by comparing them to the ground truth classification results based on the DILI pattern and severity scores.

### Robustness evaluation

The proposed DeepCausality framework employed transformer-based LMs to learn the joint probability among variables for causal inference. However, this process can be less robust due to different random seeds, even though the same hyper-parameters were chosen. Toward real-world applications, the robustness of the proposed framework was investigated based on the strategy developed in our previous study (Wang et al., 2021). Specifically, we employed the proposed DeepCausality to run parallel experiments with the same hypermeters three times. Then, the enriched causal terms in the three repeated experiments were compared using a Venn diagram and the percentage of overlapped terms (POT) strategy (Wang et al., 2021). The POT could be calculated based on two steps: (1) rank the enriched terms based on z scores from high to low in each run, and (2) calculate the POT using the number of the overlapping terms among three repeated runs divided by *L*. *L* denotes the number of enriched terms of each subset of the ranked enriched term list. In this study, *L* was set from 1 to 30 at one interval.

### Implementation of the DeepCausality

To facilitate the application of our model, we developed a standalone package for the readers' convenience. The proposed DeepCausality framework was exemplified based on a BioBERT (BioBERT, https://github.com/dmis-lab/biobert) and BERN under Python 3.6 TensorFlow version 1.15. We evaluated our proposed DeepCausality model on one NVIDIA Tesla V100 GPU. For the LiverTox dataset, the average runtime was approximately 8 h. We incorporated the Do-calculus causal function into the BioBERT source code, which easily migrated into other transformers. All the source code and the processed data sets used in this study are publicly available through https://github.com/XingqiaoWang/https-github.com-XingqiaoWang-DeepCausality-LiverTox.

## Results

### Data preprocessing of the LiverTox dataset

**Figure 2:**
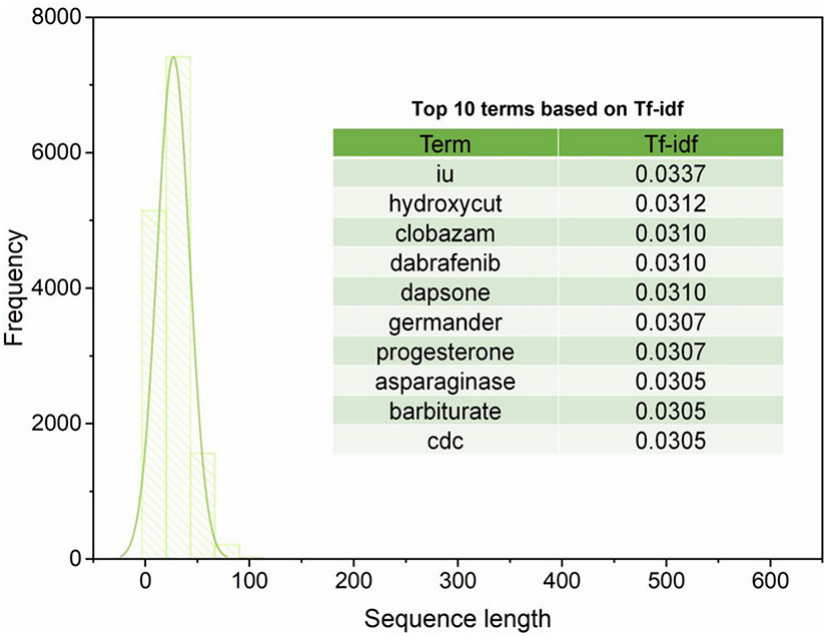
The distribution of sequence length and the top 10 terms based on Tf-idf values.

Figure 2 illustrates the sequence length of the extracted 14,361 sentences from four sections (i.e., Introduction, Background, Hepatotoxicity, Mechanism of Liver Injury) of LiverTox. The average and standard deviation of the sequence length of the extracted 14,361 sentences is 26.84 ± 15.58. Furthermore, the extracted 14,361 sentences contain 15,804 unique words (Supplementary Table S1). We observed the top ten terms based on the term frequency-inverse document frequency (Tf-idf) values, including *iu, hydroxycut, clobazam, dabrafenib, dapsone, germander, progesterone, asparaginase, barbiturate*, and *CDC*. These top ten terms were not directly associated with any current knowledge of iDILI, indicating the causal factors could not be enriched by the simple frequency-based strategy.

### Fine-tune BioBERT model with LiverTox data

Considering that LiverTox is summarized from literature and clinical reports, we employed BioBERT to establish the joint probability between variables. For that, we divided the extracted 14,361 sentences into two sets with a ratio of 9:1 in a stratified manner, with the ratio between positives (i.e., iDILI positives) and negatives (i.e., iDILI negatives) kept constant for both sets. It resulted in 12,924 (14,361 × 90% = 12,924) and 1,437 (14,361 × 10% = 1,437) sentences in training and test sets, respectively (Table 1). Then, we employed BioBERT-Base v1.1 (+ PubMed 1M), consisting of 12 transformer layers, 128 embeddings, 768 hidden, and 12 heads with 11M parameters. We further fine-tuned the BioBERT_base_ model with the 12,924 sentences in the training set. We determined the optimized models based on the text classification result in the test set for iDILI sentence prediction. Specifically, we set the maximum sequence length to 128 and the mini-batch size to 128. A total of 2,500 training steps were implemented with a 500-step warmup, and the checkpoint step was set to 200 for recording the prediction results.

**Table 1:** Data information of preprocessed sentences in LiverTox.

**Dataset**	**iDILI positive**	**iDILI negative**	**Positive ratio**	**Total**
Training set	3,218	9,706	0.249	12,924
Test set	360	1,077	0.251	1,437
Total	3,578	10,783	0.249	14,361

**Figure 3:**
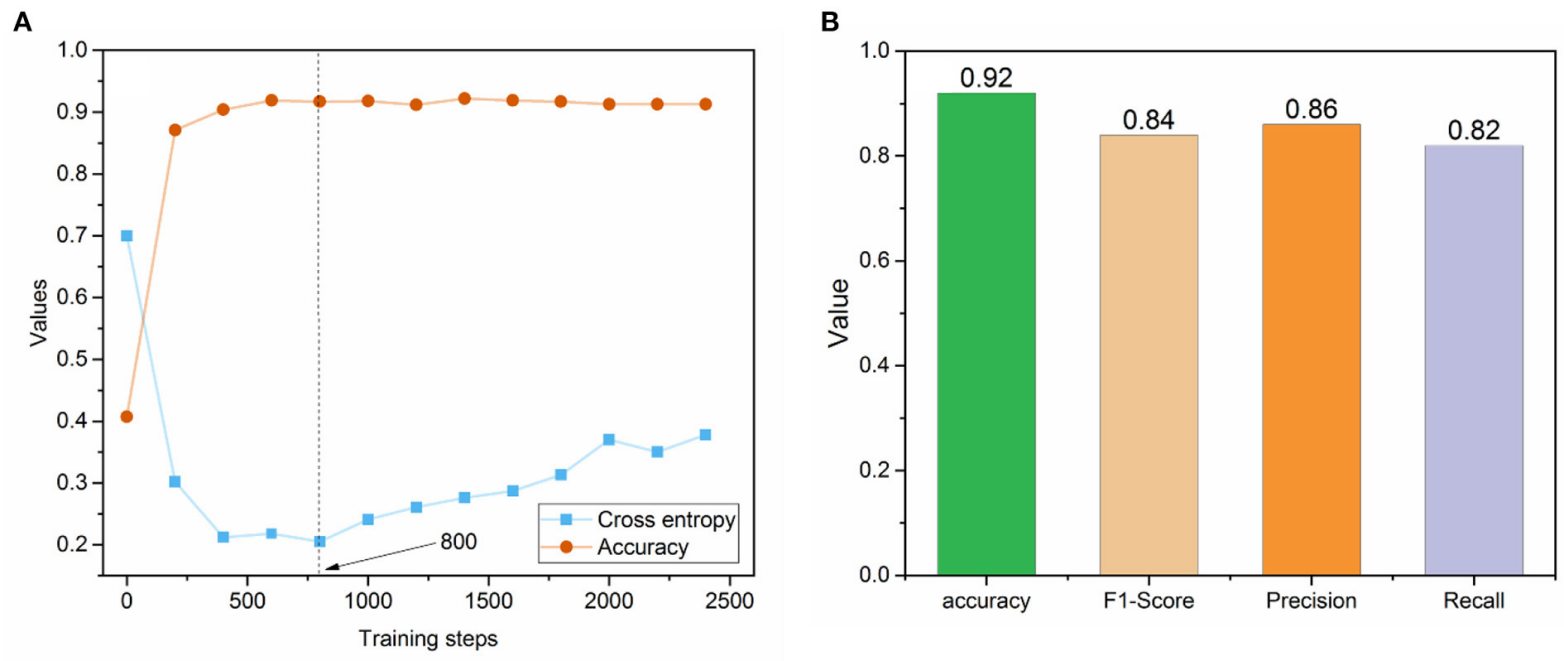
**(A)** The trend of cross-entropy loss and accuracy across the different training steps in the fine-tuned BioBERT model; **(B)** Prediction performance metrics of the optimized fine-tuned BioBERT model on the test set.

Figure 3A depicted the trends of cross entropy loss and accuracy while increasing the number of training steps based on the text set. The cross-entropy loss decreased dramatically before 400 training steps and became stable between 400 and 800 training steps. Then, it increased after 1,000 steps, indicating the potential of overfitting phenomena. Meanwhile, the accuracies of the dataset tended to be stable after training step 400. Thus, we selected the optimized fine-tuned model based on the training step with the minimum loss (i.e., 800), where the accuracy value also showed no dramatic changes. The optimized fine-tuned model yielded a high accuracy of 0.92, an F1-score of 0.84, a precision of 0.86, and a recall of 0.82 in the test set, indicating the optimized fine-tuned model well captured the relationship between variables (Figure 3B).

### Biomedical-based named entity recognition

To carry out the causal inference within the biomedical-based NER terms, we employed the BERN to extract the biomedical-related terms from the preprocessed sentences. We obtained a total of 87 biomedical-related terms that were divided into three categories using BERN, including 16 drugs, 11 genes, and 60 diseases (see Supplementary Table S2). Through the biomedical-based NER, we narrowed down the total terms (unique words) in the preprocessed sentences from a total of 15,804 to 87, with a 99.4% compression rate.

### Causal inference using NER-based Do-calculus

To further investigate whether the performance of the proposed DeepCausality could identify the causal terms of iDILI, we implemented NER-based Do-calculus to uncover the predictors from the fine-tuned BioBERT model (Table 2). Of 87 Biomedical-based name entities, 24 name entities were enriched with an adjusted *p* value < 0.05 based on a one-tail z-test using the NER-based Do-calculus. We excluded 4 drug entities, including iron, isoniazid, rifampin, and acetaminophen, since our objective is to identify the causal factors related to iDILI. For example, acetaminophen is a protype drug for dose-dependent drug-induced liver injury (DILI), which is not idiosyncratic in nature (Jaeschke, 2015). Furthermore, 18 of 20 enriched causal terms were highly consistent with current knowledge of iDILI, yielding an enrichment rate of 90% (Chalasani et al., 2021). These name entities were distributed into different categories, including Liver Enzymes, Concomitant diseases, History of other liver disorders, Physical findings, Laboratory results, Symptoms and Signs, and Clinical outcomes based on the ACG clinical guideline for iDILI diagnosis.

**Table 2:** Causal inference results for idiosyncratic DILI.

**Elements**	**Z score**	**Probability of DO value**	**Probability of not DO value**	**Probability difference**
**Liver Enzymes**				
Alkaline phosphatase	3.772	0.398	0.244	0.154
ALT	2.561	0.307	0.244	0.064
**Concomitant diseases**				
Tuberculosis	2.470	0.382	0.244	0.138
Rheumatoid arthritis	1.759	0.334	0.244	0.089
**History of other liver disorder**				
Cholestasis	4.827	0.547	0.244	0.303
Cholestatic hepatitis	3.653	0.499	0.244	0.255
**Physical findings**				
Fever	6.508	0.383	0.241	0.141
Pain	2.377	0.395	0.244	0.150
**Laboratory results**				
Lactic acidosis	3.181	0.460	0.244	0.216
**Symptoms and signs**				
Hypersensitivity	3.966	0.383	0.243	0.139
Skin rash	2.066	0.333	0.244	0.088
Jaundice	1.773	0.274	0.244	0.030
Stevens Johnson syndrome	1.669	0.335	0.245	0.090
**Clinical outcome**				
Hepatic failure	4.119	0.437	0.244	0.193
Cirrhosis	2.944	0.391	0.244	0.147
Liver failure	2.905	0.366	0.244	0.122
Sinusoidal obstruction syndrome	2.490	0.403	0.244	0.159
Acute liver failure	1.669	0.326	0.244	0.082

Table 2 lists enriched causal factors ranked based on the Z score. The causal factor (Z score) are as follows: for Liver Enzymes, alkaline phosphatase (3.772), ALT (2.561); for Concomitant diseases, tuberculosis (2.470), rheumatoid arthritis (1.759); for History of other liver disorder, cholestasis (4.827), cholestatic hepatitis (3.653); for Physical findings, fever (6.508), pain (2.377); for Laboratory results, lactic acidosis (3.181); for Symptoms and Signs, hypersensitivity (3.966), skin rash (2.066), jaundice (1.773), and Stevens-Johnson syndrome (1.669); for Clinical outcome, hepatic failure (4.119), cirrhosis (2.944), liver failure (2.905), sinusoidal obstruction syndrome (2.490), and acute liver failure (1.669).

**Figure 4:**
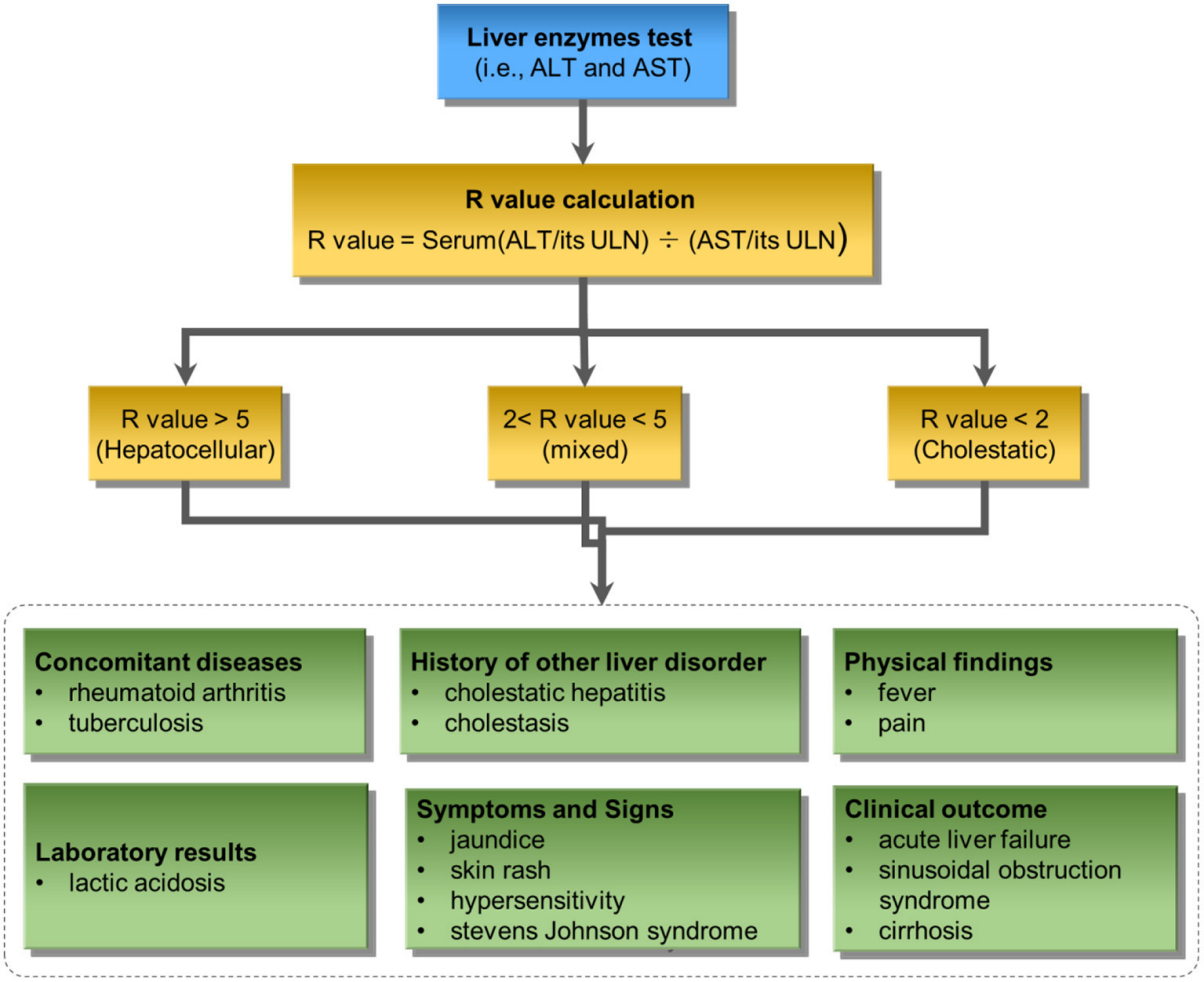
The proposed knowledge-based causal tree based on the ACG clinical guideline on iDILI patient diagnosis: ULN denotes upper limits of normal.

Figure 4 illustrates the developed knowledge-based causal tree with enriched causal factors based on the ACG clinical guideline for iDILI diagnosis. The proposed knowledge-based prediction tree could be divided into two major components: liver enzyme test and clinical observations. The liver enzyme test, including ALT and AST, divides iDILI patients into different DILI patterns, including hepatocellular, mixed, and cholestatic. Clinical observations could further classify the iDILI patients based on their severity and clinical symptoms.

### iDILI patient stratification

**Figure 5:**
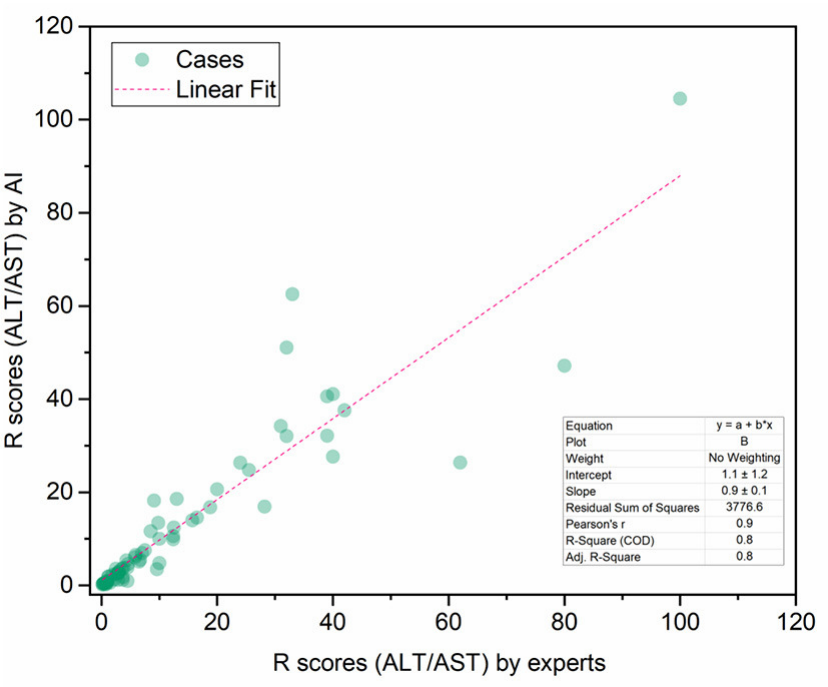
The correction between the R scores (ALT/AST) calculated by DeepCausality and expert: ALT and AST stand for Alanine transaminase and aspartate transaminase, respectively.

**Figure 6:**
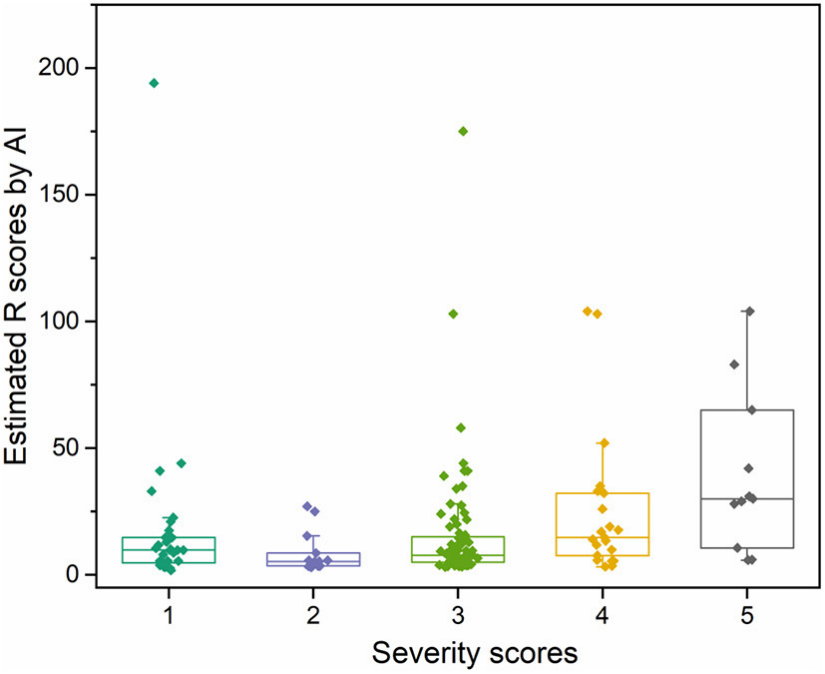
The distribution of iDILI patients stratified by DeepCausality across the different severity levels defined by domain experts.

To demonstrate the proposed knowledge-based causal tree could be utilized for iDILI patient stratification, we stratified 175 patients' case reports in the LiverTox dataset based on the developed causal tree and compared expert-based patient stratification results. There was a high correlation between the R (ALT/AST) values determined by DeepCausality and the experts, with a Pearson correlation coefficient of more than 0.9 (Figure 5). Furthermore, we observed that the clinical observations in the developed causal tree could be used to classify the patients into different severity groups, distinguished by the R scores estimated by DeepCausality (Figure 6).

### Robustness of DeepCausality

**Figure 7:**
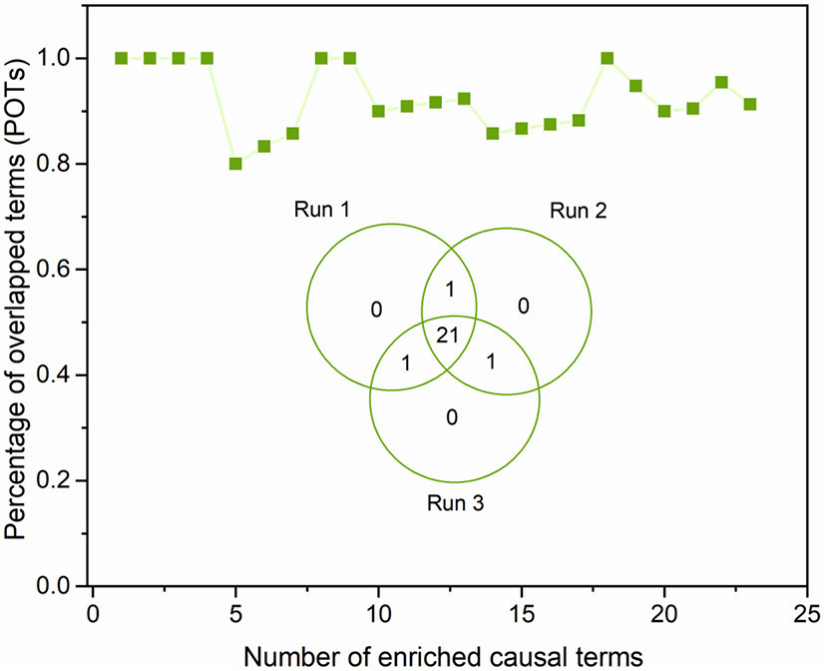
Robustness evaluation of the proposed DeepCausality: The Venn diagram illustrates the overlapping of the enriched causal terms by three parallel runs. The dotted-line curve illustrates the percentage of overlapping causal terms (POTs) among the three repeated runs across ranked order terms by z scores.

To ensure the proposed DeepCausality could generate reproducible causal inference results, we investigated the robustness of causal inference results by running the DeepCausality three times (see Supplementary Table S3). Figure 7 depicted the POT enrichment after three different runs. We found highly reproducible results from three parallel runs of DeepCausality, with an average POT of 0.923. Furthermore, the Venn diagram indicates 87.5% commonality of enriched causal terms after three runs. Altogether, the proposed DeepCausality framework could generate highly repeatable results without interfering with factors such as initial seeds.

## Discussion

Causality is one of the most critical notions in every branch of science. Causal inference based on observational data has gained more and more momentum as an alternative to the conventional random controlled trial-based causality assessment. Notably, More and more advocates promote using RWD and RWE to monitor post-market safety and adverse events and make regulatory decisions in drug development. An essential resource of RWD, observational data such as EHRs, clinical reports, and patient narratives are typically free text-based, posing a significant challenge to uncovering hidden causal factors. AI-powered LMs such as transformers have shown great potential in various NLP tasks such as text classification, information retrieval, question & answering, and sentimental analysis. However, leveraging these AI-powered LMs to conduct causal inference as a human does is still at the infant stage. To bridge this gap, we proposed DeepCausality, a general AI-powered causal inference framework for free text. We exemplified the utility of the proposed DeepCausality for iDILI-related causal factor identification based on LiverTox and applied it to iDILI patient stratification. Consequently, DeepCausality identified 20 causal factors for iDILI, and 18 (90%) were aligned with the current clinical knowledge of iDILI. Furthermore, the developed knowledge-based causal tree was used to classify iDILI patients, which was highly consistent with stratification results based on domain experts.

AI-based language models such as transformers rely on a pre-trained model with a large corpus and then use the learned knowledge to solve the downstream tasks. In this study, without training on a large number of DILI-related literature and clinical reports, we hypothesized the accumulated knowledge from these large corpora of documents could be an alternative to accelerate the training process of transformer-based LMs. Furthermore, we introduced the domain-specific named entity recognition (NER) step into the general framework, aiming to eliminate the false positives and irrelevant enrichment in the causal inference process. If available, this step could also be substituted with domain-specific ontology and knowledge graphs.

One of the initial attempts conveyed in this study was to use the developed knowledge-based causal tree for iDILI patient stratification. The high consistency of iDILI patient stratification results from DeepCausality with determination by experts is encouraging. However, it is worth pointing out the causal tree was developed based on prior knowledge of iDILI diagnosis, indicating that expert knowledge is still an indispensable component to facilitating AI-based approaches in real-world applications.

It is also worth investigating a few aspects of the proposed DeepCausality for potential improvements. In this study, to showcase the proposed DeepCausality, we employed a biomedical-based free text in LiverTox. Additional validation of the utility in other domains is highly recommended. To facilitate the process, all developed codes, scripts, and processed datasets are open to the public through https://github.com/XingqiaoWang/https-github.com-XingqiaoWang-DeepCausality-LiverTox. Additionally, the BERT-based model was incorporated into the DeepCausality framework presented here. Some generative-based transformers, such as Generative Pre-trained Transformer 3 (GPT3), do not need intensive task-specific training (Brown et al., 2020), which may be a more efficient way to conduct causal inference. Lastly, although DeepCausality could identify the causal factors, it could not classify the identified causal factors further into cofounders or colliders. It may be solved by developing directional DO-calculus statistics in the Bayesian networks derived from the transformers.

In conclusion, DeepCausality provided an AI-powered solution for causal inference in free text by integrating transformers, NER, and Do-calculus into a unified framework. DeepCausality is proposed for real-world applications to promote RWE collection and utilization.

## Data availability statement

The original contributions presented in the study are included in the article/Supplementary material, further inquiries can be directed to the corresponding author/s.

## Author contributions

XX devised the DeepCausality model applied in this study. ZL and WT conceived and designed the study and the utilization of the LiverTox database. XW coded the DeepCausality model. XW and ZL performed data analysis. ZL, XW, and XX wrote the manuscript. WT and QL revised the manuscript. All authors read and approved the final manuscript.

## Conflict of interest

The authors declare that the research was conducted in the absence of any commercial or financial relationships that could be construed as a potential conflict of interest.

## Publisher's note

All claims expressed in this article are solely those of the authors and do not necessarily represent those of their affiliated organizations, or those of the publisher, the editors and the reviewers. Any product that may be evaluated in this article, or claim that may be made by its manufacturer, is not guaranteed or endorsed by the publisher.

## Author disclaimer

This manuscript reflects the views of the authors and does not necessarily reflect those of the Food and Drug Administration. Any mention of commercial products is for clarification only and is not intended as approval, endorsement, or recommendation.

## References

[B1] Beltagy I. Lo K. (2019). SciBERT: A pretrained language model for scientific text. arXiv [Preprint]. arXiv:1903.10676. 10.18653/v1/D19-137135062081

[B2] Brown T. Mann B. Ryder N. Subbiah M. Kaplan J. D. Dhariwal P.. (2020). Language models are few-shot learners. Adv. Neural Inf. Process. Syst. 33, 1877–1901. 10.48550/arXiv.2005.14165

[B3] Chalasani N. P. Maddur H. Russo M. W. Wong R. J. (2021). ACG clinical guideline: diagnosis and management of idiosyncratic drug-induced liver injury. ACG 116, 878–898. 10.14309/ajg.0000000000001259 33929376

[B4] Chalkidis I. Fergadiotis M. Malakasiotis P. Aletras N. (2020). LEGAL-BERT: The muppets straight out of law school. arXiv [Preprint]. arXiv:2010.02559.

[B5] Clark K. Luong M. T. Le Q. V. (2020). Electra: Pre-training text encoders as discriminators rather than generators. arXiv [Preprint]. arXiv:2003.10555.34330259

[B6] Devlin J. Chang M. W. Lee K. (2018). Bert: Pretraining of deep bidirectional transformers for language understanding. arXiv [Preprint]. arXiv:1810.04805.32723719

[B7] Frieden T. R. (2017). Evidence for health decision making — beyond randomized, controlled trials. New Engl. J. Med. 377, 465–475. 10.1056/NEJMra1614394 28767357

[B8] Gajra A. Zettler M. E. Feinberg B. A. (2020). Randomization versus Real-World Evidence. New England J. Med. 383, e21. 10.1056/NEJMc2020020 32706546

[B9] Gu Y. Tinn R. Cheng H. Lucas M. Usuyama N. Liu X.. (2021). Domain-specific language model pretraining for biomedical natural language processing. ACM Trans. Comput. Healthcare (HEALTH) 3, 1–23. 10.1145/3458754

[B10] Hannun A. Case C. Casper J. Catanzaro B. Diamos G. Elsen E.. (2014). Deep speech: Scaling up end-to-end speech recognition. arXiv [Preprint]. arXiv:1412.5567.

[B11] Hernán M. A. (2021). Methods of public health research — strengthening causal inference from observational data. New Engl. J. Med. 385, 1345–1348. 10.1056/NEJMp2113319 34596980

[B12] Ho M. van der Laan M. Lee H. Chen J. Lee K. Fang Y.. (2021). The current landscape in biostatistics of real-world data and evidence: causal inference frameworks for study design and analysis. Stat. Biopharmaceut. Res. 52, 511–525. 10.1080/19466315.2021.1883475

[B13] Hoofnagle J. H. (2013). LiverTox: a website on drug-induced liver injury,” in Drug-Induced Liver Disease (Elsevier) 725–732. 10.1016/B978-0-12-387817-5.00040-6 23456678 PMC5044298

[B14] Huang K. Altosaar J. (2019). Clinicalbert: Modeling clinical notes and predicting hospital readmission. arXiv [Preprint]. arXiv:1904.05342.

[B15] Jaeschke H. (2015). Acetaminophen: Dose-dependent drug hepatotoxicity and acute liver failure in patients. Dig. Dis. 33, 464–471. 10.1159/000374090 26159260 PMC4520394

[B16] Kim D. Lee J. So C. H. Jeon H. Jeong M. Choi Y.. (2019). A neural named entity recognition and multi-type normalization tool for biomedical text mining. IEEE Access 7, 73729–73740. 10.1109/ACCESS.2019.292070830666476

[B17] Lan Z. Chen M. Goodman S. Gimpel K. Sharma P. Soricut R.. (2019). Albert: A lite bert for self-supervised learning of language representations. arXiv [Preprint]. arXiv:1909.11942.

[B18] Lee J. Yoon W. Kim S. Kim D. Kim S. So C. H.. (2019). BioBERT: a pre-trained biomedical language representation model for biomedical text mining. Bioinformatics 36, 1234–1240. 31501885 10.1093/bioinformatics/btz682 PMC7703786

[B19] Lee J. Yoon W. Kim S. Kim D. Kim S. So C. H.. (2020). BioBERT: a pre-trained biomedical language representation model for biomedical text mining. Bioinformatics 36, 1234–1240. 10.1093/bioinformatics/btz682 31501885 PMC7703786

[B20] Liu Y. Ott M. Goyal N. Du J. Joshi M. Chen D.. (2019). Roberta: A robustly optimized bert pretraining approach. arXiv [Preprint]. arXiv:1907.11692.

[B21] Liu Z. Roberts R. A. Lal-Nag M. Chen X. Huang R. Tong W.. (2021). AI-based language models powering drug discovery and development. Drug Discov. Today 26, 2593–2607. 10.1016/j.drudis.2021.06.009 34216835 PMC8604259

[B22] Mazhar H. Foster B. C. Necyk C. Gardiner P. M. Harris C. S. Robaey P.. (2020). Natural health product-drug interaction causality assessment in pediatric adverse event reports associated with attention-deficit/hyperactivity disorder medication. J. Child Adolesc. Psychopharmacol. 30, 38–47. 10.1089/cap.2019.0102 31670573

[B23] Naidu R. P. (2013). Causality assessment: A brief insight into practices in pharmaceutical industry. Perspect. Clin. Res. 4, 233–236. 10.4103/2229-3485.120173 24312892 PMC3835968

[B24] O'Mahony N. Campbell S. Carvalho A. Harapanahalli S. Hernandez G. V. Krpalkova L.. (2019). Deep learning vs. traditional computer vision,” in Science and Information Conference (Springer) 128–144. 10.1007/978-3-030-17795-9_10

[B25] Pearl J. (2009). Causality: Models, Reasoning and Inference. Cambridge: Cambridge University Press. 10.1017/CBO9780511803161

[B26] Pearl J. Mackenzie D. (2018). The Book of Why: The New Science of Cause and Effect. Penguin: Basic Books.

[B27] Sanh V. Debut L. Chaumond J. (2019). DistilBERT, a distilled version of BERT: smaller, faster, cheaper and lighter. arXiv [Preprint]. arXiv:1910.01108.

[B28] Schölkopf B. (2019). Causality for machine learning. arXiv [Preprint]. arXiv:1911.10500.

[B29] Shrestha Y. R. Ben-Menahem S. M. Von Krogh G. (2019). Organizational decision-making structures in the age of artificial intelligence. California Manag. Rev. 61, 66–83. 10.1177/0008125619862257

[B30] Tucci R. R. (2013). Introduction to Judea Pearl's Do-Calculus. arXiv [Preprint]. arXiv:1305.5506.

[B31] Veitch V. Sridhar D. Blei D. (2020). “Adapting text embeddings for causal inference,” in Conference on Uncertainty in Artificial Intelligence, PMLR, 919–928.

[B32] Wang X. Xu X. Tong W. Roberts R. Liu Z. (2021). InferBERT: A transformer-based causal inference framework for enhancing pharmacovigilance. Front. Artific. Intell. 4, 659622. 10.3389/frai.2021.659622 34136800 PMC8202286

[B33] Zheng C. Dai R. Gale R. P. Zhang M. J. (2020). Causal inference in randomized clinical trials. Bone Marrow Transpl. 55, 4–8. 10.1038/s41409-018-0424-x 30914756

